# Extracellular Vesicles and Diabetes Research: Current Status and Future Promise

**DOI:** 10.3390/biom16060909

**Published:** 2026-06-19

**Authors:** Mohamed S. Gad, Samar Habib, Khaled Elmasry

**Affiliations:** 1 Department of Ophthalmology, Medical College of Georgia, Augusta University, Augusta, GA 30912, USA; mgad@augusta.edu; 2 Department of Medical Histology and Cell Biology, Faculty of Medicine, Mansoura University, Mansoura 35516, Egypt; 3 Department of Medical Parasitology, Faculty of Medicine, Mansoura University, Mansoura 35516, Egypt; dr_samarhabib@mans.edu.eg; 4 Department of Biomedical Sciences, College of Medicine, Dubai Medical University, Dubai 20170, United Arab Emirates

**Keywords:** extracellular vesicles, exosomes, diabetes, diabetic retinopathy, diabetic nephropathy

## Abstract

Diabetes mellitus represents a major global health challenge with rapidly increasing prevalence and substantial morbidity driven by metabolic and vascular complications. Extracellular vesicles (EVs) have emerged as critical mediators of intercellular communication and are increasingly implicated in the pathogenesis and progression of diabetes. This review summarizes current knowledge on EV biology, including their classification, cellular sources, biogenesis, uptake mechanisms, and molecular cargo. We discuss the contribution of EV-associated microRNAs to immune dysregulation and β-cell damage in type 1 diabetes mellitus (T1DM), as well as the role of EVs in insulin resistance, metabolic signaling, and vascular dysfunction in type 2 diabetes mellitus (T2DM). Particular emphasis is placed on EV-mediated modulation of endothelial function, angiogenesis, and tissue repair, alongside their involvement in the impairment of insulin receptor integrity. We further explore how lifestyle factors may influence EV composition and function, highlighting their potential integration into preventive strategies. Finally, we evaluate the emerging therapeutic potential of EVs as biomarkers and delivery systems, while addressing current limitations and future directions. Collectively, EVs represent a promising frontier in understanding diabetes pathophysiology and developing innovative diagnostic and therapeutic approaches. Unlike previous reviews that examine EVs separately as biomarkers or therapeutic vehicles, this review integrates emerging evidence supporting EVs as mediators of systemic communication linking pancreatic islets, adipose tissue, immune cells, vascular endothelium, kidney, heart, and retina throughout diabetes progression. We further critically evaluate translational barriers that currently limit clinical implementation of EV-based diagnostics and therapeutics.

## 1. Diabetes Mellitus (DM)

### 1.1. Introduction

Diabetes mellitus (DM) comprises a heterogeneous group of chronic metabolic disorders characterized by sustained hyperglycemia, arising from impaired insulin secretion, insulin resistance, or both [1,2,3]. The principal types include type 1 diabetes mellitus (T1DM), type 2 diabetes mellitus (T2DM), and gestational diabetes mellitus (GDM), as well as rarer monogenic and secondary forms [1,2,3]. T1DM results from autoimmune destruction of pancreatic β-cells, leading to absolute insulin deficiency and commonly presenting in childhood or adolescence [4,5]. In contrast, T2DM, which represents more than 90% of cases, is defined by progressive β-cell dysfunction combined with insulin resistance and is strongly influenced by obesity, sedentary lifestyle, and genetic predisposition [6,7,8]. GDM develops during pregnancy, posing risks of adverse maternal and neonatal outcomes and increasing long-term susceptibility to T2DM for both mother and offspring [9,10]. Globally, DM affects people across all ages, genders, and regions, contributing significantly to morbidity and mortality [11,12].

### 1.2. Global Burden of DM

Data from the Global Burden of Disease 2019 study identified ischemic heart disease and stroke as the first and second leading contributors to the global disease burden, respectively, with diabetes recognized as a major predisposing factor for both conditions [13,14].

Chronic hyperglycemia associated with DM drives the development of long-term complications, broadly classified as microvascular and macrovascular [15,16]. Microvascular complications include diabetic retinopathy (DR), nephropathy, and neuropathy, which are leading causes of blindness, end-stage renal disease, and limb amputations, respectively [17,18]. Macrovascular complications, such as cardiovascular disease, stroke, and peripheral arterial disease, further exacerbate diabetes-related morbidity and mortality [15,18]. Additionally, individuals with diabetes exhibit greater susceptibility to infections, impaired wound healing, and elevated cancer risk. Collectively, these complications substantially diminish quality of life while imposing a profound healthcare and socioeconomic burden worldwide [19].

### 1.3. Epidemiology of DM

Diabetes has emerged as a global epidemic, with the International Diabetes Federation (IDF) estimating 537 million adults (20–79 years) living with the disease in 2021, a prevalence of 10.5%, and projections rising to 643 million by 2030 and 783 million (12.2%) by 2045 [2,20]. Nearly half of all cases remain undiagnosed, equating to around 240 million individuals worldwide, which delays intervention and heightens complication risks. The economic burden is immense, with global diabetes-related healthcare costs reaching $966 billion in 2021 and expected to exceed $1 trillion by 2045 [20,21].

The prevalence is disproportionately high in low- and middle-income countries, which account for nearly 80% of the global diabetic population and are projected to experience the steepest growth, largely driven by urbanization, poor nutrition, sedentary lifestyles, and poverty [22].

In 2021, nearly half of individuals with diabetes (44.7%; ~240 million) were unaware of their condition, with the highest proportions of undiagnosed cases observed in Africa (53.6%), the Western Pacific (52.8%), and Southeast Asia (51.3%). In contrast, prevalence was markedly lower in North America and the Caribbean (24.2%) [23].

## 2. Biology of EVs

### 2.1. Types and Sources of EVs

EVs are diverse, membrane-bound nanovesicles surrounded by a phospholipid bilayer and released from cells. They are produced by nearly all cell types and serve essential roles both within and between cells, including regulating physiological processes, facilitating cell-to-cell communication, transporting biomolecules, and modulating immune responses [24,25].

EVs are categorized based on various factors, such as their origin, mode of formation, size, molecular cargo, surface markers, and functional characteristics. Traditionally, they are grouped into three main types: (a) small EVs, derived from the endosomal pathway and measuring approximately 30–150 nm in diameter; (b) microvesicles, formed by outward budding of the plasma membrane and ranging from 100–1000 nm; and (c) apoptotic bodies, also released from the plasma membrane but typically larger, with sizes between 500–2000 nm [26].

### 2.2. Challenges in EV Classification

The classification of EVs remains a significant challenge in the field. According to the recommendations of the International Society for EVs and the MISEV2023 guidelines, distinguishing between different EV subtypes, particularly small EVs, microvesicles, and apoptotic bodies, is often difficult due to their overlapping size ranges, shared molecular cargo, and lack of definitive subtype-specific markers [27]. Furthermore, current isolation and characterization techniques are generally insufficient to conclusively determine the biogenesis pathway of individual vesicles, making it challenging to prove an endosomal origin—the defining feature of small EVs. As a result, the MISEV2023 recommendations encourage the use of operational terms based on physical characteristics, biochemical composition, or cell of origin rather than assuming specific biogenesis pathways [28]. Therefore, throughout this review, the term “extracellular vesicles (EVs)” is used unless an endosomal origin has been experimentally demonstrated.

### 2.3. Biogenesis of EVs

EV formation involves a coordinated process of endocytosis and exocytosis. During endocytosis, the plasma membrane invaginates, leading to the creation of intracellular multivesicular bodies (MVBs) that contain intraluminal vesicles (ILVs). Exocytosis then occurs when these ILVs are released into the extracellular space through the fusion of the MVB membrane with the plasma membrane [29,30].

**Figure 1:**
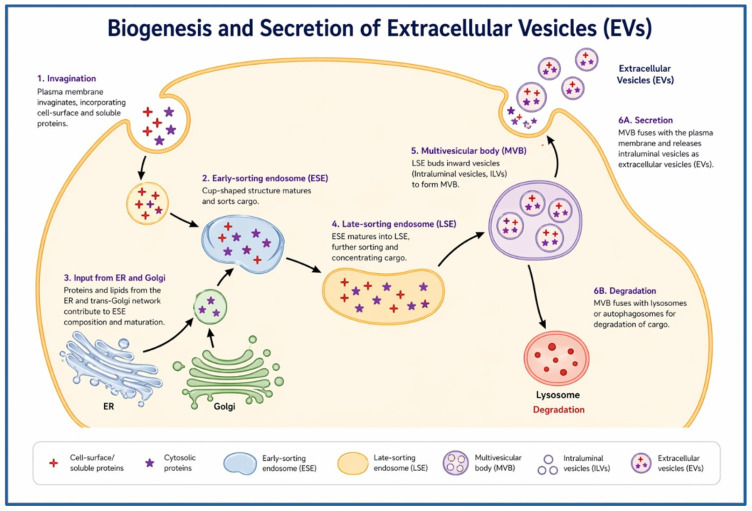
The biogenesis and secretion of EVs typically begin with the formation of a cup-shaped structure from the plasma membrane, incorporating cell-surface proteins and soluble proteins, which leads to the development of an early-sorting endosome (ESE). The trans-Golgi network and endoplasmic reticulum contribute to the ESE’s composition and maturation, after which it transitions into a late-sorting endosome (LSE) and ultimately forms a multivesicular endosome (MVB). MVBs can either fuse with lysosomes or autophagosomes for degradation, or merge with the plasma membrane, releasing their intraluminal vesicles as EVs.

The process begins with the invagination of the plasma membrane, forming a cup-shaped structure that incorporates cell-surface proteins and soluble proteins from the extracellular environment, leading to the development of an early-sorting endosome (ESE). The trans-Golgi network and endoplasmic reticulum can also contribute to the formation and molecular content of the ESE. The ESE then matures into a late-sorting endosome (LSE) and subsequently develops into a multivesicular body (MVB), also known as a multivesicular endosome. Within MVBs, inward budding of the endosomal limiting membrane generates multiple intraluminal vesicles (ILVs), which are the precursors of EVs. MVBs can either fuse with lysosomes or autophagosomes for degradation, or merge with the plasma membrane to release ILVs as EVs [30,31] (Figure 1). To ensure the selective sorting of EVs’ cargo during their formation, both endosomal sorting complex required for transport (ESCRT)-dependent and ESCRT-independent pathways (such as tetraspanin-mediated and lipid-based mechanisms) are involved. The most widely recognized pathway for generating multivesicular bodies (MVBs) and intraluminal vesicles (ILVs) is ESCRT-dependent, which involves over 20 proteins organized into four main complexes (ESCRT-0, I, II, and III) along with several associated proteins, including VPS4, VTA1, and ALIX—highly conserved from yeast to mammals. Research has also identified various bioactive molecules that regulate EV biogenesis, such as the syndecan–syntenin–ALIX complex, c-Src (a membrane-associated tyrosine kinase), the small GTPase Ral, the Atg12–Atg3 complex (linking autophagy regulation to EV formation), and mixed lineage kinase domain-like (MLKL) protein [32].

### 2.4. Uptake of EVs

Donor cells release EVs, which are taken up by recipient cells through various mechanisms to facilitate intercellular communication. EV uptake can occur in three main ways: (1) direct fusion of EVs with the plasma membrane, enabling cargo delivery to the recipient cell, although the exact mechanism remains unclear, it is thought to involve tetraspanin complexes; (2) signal transmission between EVs and target cells via receptor–ligand interactions, which may include soluble signals generated by proteolytic cleavage of EV surface ligands [33]; and (3) internalization of EVs by different endocytic pathways, such as clathrin- or caveolin-mediated endocytosis, receptor- or raft-mediated endocytosis, macropinocytosis, and phagocytosis [34,35].

### 2.5. Structure and Contents of EVs

Mirroring the structure of a cell, EVs are membrane-enclosed vesicles surrounded by a lipid bilayer that encapsulates a full complement of bioactive molecules, including proteins, nucleic acids, lipids, and carbohydrates. To date, analysis of 286 EV studies has identified 9769 proteins, 3408 mRNAs, 2838 microRNAs, and 1116 lipids, all cataloged in ExoCarta, a manually curated and comprehensive database of EV-associated cargo [36].

The EV outer membrane is primarily lipid-based and enriched with various membrane proteins. Notably, specific lipids such as cholesterol, phospholipids, and phosphatidylserine are abundant. Membrane proteins, including transmembrane and peripherally associated proteins, play critical roles in EV biological functions, while soluble luminal proteins also contribute to their activity [37]. EVs from different cellular origins share common protein components, including tetraspanins (CD63, CD9, CD81, CD82), integrins involved in cell targeting and adhesion, membrane fusion proteins linked to Rab GTPases, annexins, and flotillin, as well as molecular chaperones like heat shock proteins (HSP70 and HSP90) that mediate multivesicular body (MVB) formation [38].

**Figure 2:**
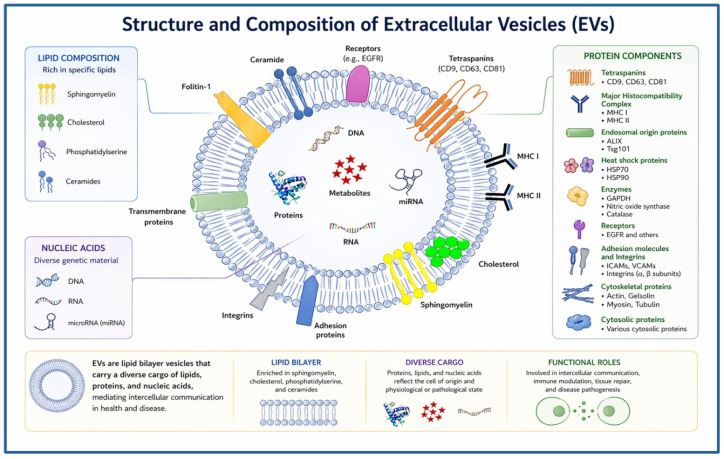
The structure and composition of EVs, as shown, reveal that they are lipid bilayer vesicles capable of carrying diverse types of cargo, including various lipids, proteins, and nucleic acids. Their membrane lipids are notably rich in sphingomyelin, phosphatidylserine, cholesterol, and ceramides. EVs also contain a heterogeneous array of proteins, such as major histocompatibility complex I and II (MHC I and MHC II), tetraspanins (CD9, CD63, CD81), endosomal origin proteins (ALIX, Tsg101), heat shock proteins (HSP70, HSP90), enzymes (GAPDH, nitric oxide synthase, catalase), receptors (EGFR), adhesion molecules, integrins, cytoskeletal proteins (actin, gelsolin, myosin, tubulin), and various cytosolic proteins. Additionally, they carry nucleic acids, including RNA, microRNA, and DNA.

Recently, EV-associated nucleic acids, particularly RNAs, have garnered attention for their roles in intercellular communication and genetic exchange. Both mRNA and microRNA (miRNA) within EVs can be transferred between cells to exert functional effects. Additionally, noncoding RNAs (ncRNAs), including miRNAs, long ncRNAs, and circular RNAs, are stably packaged within EVs and play critical roles in the pathogenesis of various diseases [39,40] (Figure 2).

### 2.6. Biological Functions Associated with EVs

EVs have been implicated in a wide range of diseases, including cancer, cardiovascular disorders, and neurological conditions. They play key roles in cell-to-cell communication, immunomodulation, tissue regeneration, cancer development, and show potential as therapeutic drug delivery vehicles. Their involvement in various ocular diseases has also been explored, though the precise mechanisms underlying these effects remain incompletely understood [41].

## 3. Are EVs Mediators, Biomarkers, or Both?

A central question in the EV field is whether EVs function merely as biomarkers that reflect underlying disease processes or whether they actively mediate disease pathogenesis. Current evidence suggests that EVs fulfill both roles; however, the strength of evidence differs substantially between mechanistic studies and clinical observations.

### 3.1. Evidence Supporting EVs as Active Mediators

In numerous experimental models, administration of EVs isolated from diseased tissues or animals reproduces pathological phenotypes in recipient cells or organisms [42]. For example, EVs derived from obese or diabetic animals can induce insulin resistance, impair glucose tolerance, promote inflammatory signaling, and alter endothelial function when transferred to healthy recipients [43]. Conversely, EVs isolated from healthy tissues or therapeutic cell populations, such as mesenchymal stromal cells (MSCs), often improve metabolic parameters, suppress inflammation, and enhance tissue repair [44]. These studies satisfy key criteria for causality by demonstrating that EVs are sufficient to transfer biological effects and that disruption of EV signaling reduces pathological outcomes.

### 3.2. Evidence Supporting EVs as Biomarkers

In contrast, most human studies provide associative rather than causal evidence. Numerous investigations have reported correlations between EV abundance, cellular origin, or molecular cargo and disease severity, progression, or therapeutic response. Altered EV concentrations and changes in EV-associated proteins, lipids, and microRNAs have been documented in diabetes, cardiovascular disease, kidney disease, retinal disorders, and other chronic conditions. These observations support the utility of EVs as accessible liquid biopsy biomarkers capable of reflecting ongoing pathological processes [45].

Clinical studies have also identified disease-specific EV signatures that correlate with metabolic dysfunction, inflammation, endothelial injury, and organ-specific complications. However, because these studies are largely observational, they cannot establish whether EVs are drivers of disease or simply consequences of tissue injury and cellular stress. The presence of strong clinical correlations therefore supports biomarker utility but does not, by itself, demonstrate mechanistic involvement.

Taken together, the available evidence supports a dual role for EVs as both mediators and biomarkers of disease.

## 4. Methods for EV Isolation and Purification

Achieving absolute pure isolation of EVs remains a challenge. Most existing protocols primarily separate small EVs by removing larger vesicles, without definitive confirmation of their cellular origin. A variety of methods have been developed that exploit the unique properties of EVs, each offering distinct advantages and limitations. Common approaches include differential ultracentrifugation, density-gradient separation, size exclusion chromatography, immunoaffinity-based isolation, and polymer-based precipitation. In practice, EV purification often relies on a combination of these techniques [46,47].

(a) Differential Ultracentrifugation

Differential ultracentrifugation is the most widely applied approach for EV isolation. By subjecting samples to increasing centrifugal forces, larger components such as dead cells, debris, and apoptotic bodies are sequentially removed, leaving smaller vesicles like EVs in the supernatant. Although widely adopted, this method is labor-intensive, time-consuming, and prone to contamination. Isolation typically requires multiple cycles at different speeds and durations, all performed at 4 °C [48,49].

(b) Density-Gradient Separation

When combined with ultracentrifugation, density-gradient separation improves purity by exploiting differences in buoyant density. Sucrose and iodixanol are commonly employed media for this method. Beyond enhancing purity, density-gradient techniques help preserve the physical integrity and biological activity of EVs more effectively than ultracentrifugation alone [48,49].

(c) Size Exclusion Chromatography

Size Exclusion Chromatography (SEC) is particularly suitable for isolating EVs from plasma. In this method, samples are passed through a porous polymer column that separates particles based on size: larger particles elute first, followed by smaller ones, including EVs. Compared with ultracentrifugation, SEC offers a gentler and more reproducible isolation process [50,51].

(d) Immunological Isolation

Immunoaffinity capture (IAC) is a highly specific approach that leverages antibodies against EVs’ surface markers. Typically, EVs are incubated with antibody-coated magnetic beads, enabling selective binding and recovery of targeted EV subpopulations [48,52].

(e) Precipitation

Precipitation methods employ polymers such as polyethylene glycol (PEG) to isolate EVs. PEG induces aggregation by encapsulating large numbers of vesicles, which can then be separated from the supernatant. This approach is simple and widely used for isolating EVs from cell culture media, although it often co-precipitates non-EVs’ contaminants [52] (Table 1).

Emerging technologies including microfluidic platforms, tangential flow filtration (TFF), acoustic separation systems, and asymmetric flow field-flow fractionation (AF4) have recently gained attention because of their improved scalability, automation, purity, and suitability for clinical-grade EV production [53]. These methods may facilitate future standardization and large-scale manufacturing of EV-based diagnostics and therapeutics.

## 5. Techniques for EV Quantification

Several approaches are currently employed for EV quantification, but given the relative novelty of the field, further comparative studies are needed to assess their accuracy and reproducibility [54].

(a) Nanoparticle tracking analysis (NTA)

Originally developed in 2003, NTA has become one of the most widely used methods for EV quantification. The technique measures the Brownian motion of particles in suspension and relates it to their hydrodynamic size using the Stokes–Einstein equation. It enables visualization and size distribution profiling of particles ranging from ~10–1000 nm. Unlike ensemble methods such as dynamic light scattering, NTA analyzes particles individually, allowing more precise characterization and even monitoring of processes such as aggregation or dissolution. Advantages include minimal sample preparation and real-time imaging; however, detection at the lower size range requires particles with high refractive indices, while accuracy at the upper size limit is reduced due to slower Brownian motion and solvent viscosity effects [55,56,57,58,59,60].

(b) Vesicle Flow Cytometry

Flow cytometry detects particles in fluid suspensions by measuring light scatter and fluorescence as they pass through a laser beam. For EV analysis, instruments are either adapted from conventional cell-based cytometers or custom-built with enhanced sensitivity, including reduced flow rates, smaller probe volumes, and modified optics. Despite its utility, flow cytometry has limited sensitivity for the smallest vesicles, often skewing size distribution towards larger particles. Moreover, variations in optical properties complicate the correlation between particle size and detected signal [61,62,63].

(c) Surface Plasmon Resonance

Surface Plasmon Resonance (SPR) is an emerging, though not yet commercially available, method for EV quantification. It works by detecting binding events between EVs and functionalized surfaces, often using antibodies such as those targeting CD63. While sensitive, this reliance on binding may interfere with accurate particle quantification, making standardization difficult [60,64].

(d) Electron Microscopy (EM)

Electron microscopy was the earliest technique used to identify EVs and has served as a benchmark for confirming sample quality. However, it is no longer commonly employed for quantification due to significant drawbacks. Standard EM sample preparation methods, such as dehydration and embedding, can cause EV loss and structural changes, leading to an underestimation of vesicle numbers [48] (Table 2).

Recent advances in EV characterization include nano-flow cytometry (nFCM) [65], tunable resistive pulse sensing (TRPS) [66], single-particle interferometric reflectance imaging sensing (SP-IRIS/ExoView), imaging flow cytometry, and microfluidic-based analytical platforms [67]. These technologies offer improved sensitivity for small EV detection, single-particle characterization, multiplex phenotyping, and higher analytical reproducibility.

## 6. EVs and Type 1 Diabetes Mellitus

Type 1 diabetes mellitus (T1DM) is a multifactorial autoimmune disease resulting from the destruction of pancreatic β-cells through interactions among genetic susceptibility, environmental factors, infectious triggers, and immune dysregulation [68]. Growing evidence identifies EVs as important mediators of intercellular communication in T1DM, influencing β-cell survival, immune activation, inflammatory signaling, and tissue repair through the transfer of proteins, lipids, and nucleic acids [45,69]. Consequently, EVs have emerged as both contributors to disease pathogenesis and promising candidates for diagnostic and therapeutic applications.

### 6.1. Experimental Evidence

#### 6.1.1. NOD Mouse Models

The non-obese diabetic (NOD) mouse remains the most widely used spontaneous model of autoimmune T1DM and has provided substantial evidence for EV involvement in disease initiation and progression. β-cell-derived EVs released during cellular stress contain diabetes-associated autoantigens, including GAD65, ZnT8, GLUT2, and proinsulin, which can be internalized by antigen-presenting cells and subsequently activate autoreactive T cells [70,71]. These EVs selectively stimulate memory T cells from patients with T1DM and contribute to the amplification of autoimmune responses through activation of Toll-like receptor (TLR) signaling and pro-inflammatory cytokine production [69,71].

Several studies have demonstrated that EVs exhibit both pathogenic and protective immunological functions in NOD mice. Macrophage-derived EVs promote inflammatory cytokine release and T-cell activation, thereby accelerating autoimmune destruction of β-cells [72]. In contrast, mesenchymal stromal cell (MSC)-derived EVs suppress antigen-presenting cell activation, inhibit Th1 and Th17 responses, enhance regulatory T-cell (Treg) expansion, and delay diabetes onset [73,74,75]. Furthermore, MSC-derived EVs increase IL-10 and TGF-β secretion while reducing pro-inflammatory cytokine production, promoting immune tolerance and preservation of β-cell function [76,77,78,79].

#### 6.1.2. Streptozotocin (STZ) Models

Chemically induced diabetic models generated by streptozotocin (STZ) administration have provided important insights into the regenerative and protective functions of EVs. Bone marrow transplantation in diabetic mice stimulates the release of EVs enriched with miR-106b-5p and miR-222-3p, which target negative regulators of the cell cycle and promote β-cell proliferation [80]. Similarly, umbilical cord MSC-derived EVs reduce endoplasmic reticulum stress, increase HIF-1α expression, and deliver VEGF, thereby enhancing β-cell survival and function under diabetic and hypoxic conditions [81].

Studies in STZ-induced diabetes have also demonstrated the therapeutic potential of EVs for islet transplantation. Human bone marrow stromal cell-derived EVs carrying anti-Fas siRNA and miR-375 inhibitors protect transplanted islets from apoptosis and improve graft function [82]. Additionally, EV-mediated delivery of angiogenic factors promotes revascularization of transplanted islets, a critical determinant of long-term graft survival [83,84].

#### 6.1.3. Cell Culture Studies

In vitro studies have further elucidated the molecular mechanisms through which EVs influence β-cell biology and immune responses. Exposure of β-cells to hyperglycemia or inflammatory cytokines increases EV secretion and alters EV cargo composition, enhancing their immunostimulatory properties [70]. Insulinoma-derived EVs activate MyD88-dependent inflammatory pathways and stimulate cytokine production, contributing to T-cell proliferation and autoimmune activation [72].

EVs have also been implicated in β-cell differentiation and regeneration. β-cell-derived EVs enriched in miR-212/132 promote differentiation of induced pluripotent stem cells (iPSCs) into insulin-producing β-cells by suppressing FBW7, restoring NGN3 expression, and activating PDX1 signaling pathways [85]. These findings suggest that EVs may play important roles in β-cell replacement strategies and regenerative therapies for T1DM.

### 6.2. Clinical Evidence

#### 6.2.1. Patient Plasma EVs

Clinical studies have revealed significant alterations in circulating EV populations in individuals with T1DM. Compared with healthy controls, patients with T1DM exhibit reductions in both EV size and concentration, indicating disease-associated changes in EV biogenesis and release [86]. Furthermore, circulating EVs carry diabetes-related autoantigens, including GAD65, IA-2, IRS1, and proinsulin, supporting their potential role in ongoing autoimmune activity and disease monitoring [87,88].

Current T1DM risk assessment relies primarily on HLA genotyping and detection of islet autoantibodies such as GAD65, IA-2, insulin, and ZnT8 [70]. However, these markers have important limitations, as autoantibodies often appear after substantial β-cell destruction has already occurred and do not consistently predict disease progression [89,90]. Consequently, circulating EVs have attracted increasing interest as alternative biomarkers capable of providing earlier insights into disease development.

#### 6.2.2. EV Biomarkers

Among the most promising clinical applications of EVs is their use as biomarker carriers. Disease-associated alterations in EV cargo, particularly EV-associated microRNAs (miRNAs), have been linked to β-cell dysfunction, immune activation, and disease progression. Several miRNAs, including miR-21, miR-29, miR-34a, and miR-146a, contribute to β-cell dysfunction and inflammatory responses, whereas miR-375 and miR-148a-3p promote β-cell death [86,91,92].

Notably, miR-375 levels are elevated in circulating EVs up to two weeks before diabetes onset in experimental models, suggesting potential utility as an early indicator of β-cell injury [93]. In newly diagnosed patients with T1DM, EV miRNA profiles demonstrate downregulation of miR-195 and miR-455 and upregulation of miR-185 compared with healthy controls [69,94]. Additional tissue-specific changes have been identified, including altered urinary EV miR-424 and miR-218 levels and elevated serum EV miR-21-5p [86]. Collectively, these findings support the potential of EV-associated biomarkers for early diagnosis, risk stratification, disease monitoring, and evaluation of therapeutic responses in T1DM.

## 7. EVs and Type 2 Diabetes Mellitus

Type 2 diabetes mellitus (T2DM) is a highly prevalent metabolic disorder whose incidence continues to increase alongside the global obesity epidemic. The disease is associated with substantial morbidity and mortality due to complications including hypertension, cardiovascular disease, nephropathy, and lower-extremity amputation [95]. Growing evidence indicates that EVs play important roles in the pathogenesis of T2DM by modulating insulin signaling, vascular function, inflammation, and tissue remodeling. Consequently, EVs have emerged as both potential biomarkers and therapeutic targets in T2DM.

### 7.1. Preclinical Evidence

Experimental studies have demonstrated that EVs actively contribute to the development of insulin resistance and vascular dysfunction. In vitro, EVs isolated from obese individuals impair insulin-stimulated glucose uptake, while macrophage-derived EVs disrupt GLUT4 translocation in adipocytes through downregulation of Akt signaling and activation of NF-κB pathways, thereby promoting insulin resistance [96]. Adipocyte-derived EVs further impair hepatic and skeletal muscle insulin signaling through the transfer of adipokines and regulatory molecules that interfere with glucose metabolism [97].

Recent evidence suggests that EV cargo, particularly microRNAs, plays a critical role in regulating insulin sensitivity and metabolic homeostasis [98]. Under hyperglycemic conditions, hepatocyte-derived EVs enriched in calpain-2 and γ-secretase mediate cleavage of the insulin receptor β-subunit, resulting in impaired downstream insulin signaling and persistent insulin resistance [99]. These findings indicate that EVs can directly influence the integrity of insulin receptor signaling pathways.

Beyond their metabolic effects, EVs contribute to vascular dysfunction, a hallmark of T2DM and its cardiovascular complications. EVs promote endothelial dysfunction through increased production of reactive oxygen species (ROS) generated by NADPH oxidase, mitochondria, and xanthine oxidase pathways, leading to elevated levels of superoxide and hydrogen peroxide [100]. Leukocyte-derived EVs stimulate endothelial secretion of IL-6 and IL-8, whereas T cell-derived EVs induce monocyte production of TNF-α and IL-1β and enhance leukocyte adhesion to endothelial cells. Experimental infusion of sepsis-derived EVs in mice activates NF-κB, inducible nitric oxide synthase (iNOS), and cyclooxygenase-2 (COX-2) signaling in cardiac and pulmonary tissues, further supporting a direct pro-inflammatory role for EVs [69].

Interestingly, EVs may also exert protective and reparative functions. Endothelial- and platelet-derived EVs can promote angiogenesis through the delivery of vascular endothelial growth factor A (VEGF-A) and endothelial nitric oxide synthase (eNOS), enhancing endothelial repair, blood flow, and tissue perfusion [101]. These adaptive responses may indirectly improve insulin-mediated glucose uptake in skeletal muscle, highlighting the dual pathogenic and protective roles of EVs in metabolic disease.

Animal and mechanistic studies further suggest that lifestyle interventions may modify EV biology. Exercise has been proposed to alter EV release and cargo composition, reducing circulating glucose, lipid levels, and inflammatory mediators while improving endothelial function and insulin sensitivity. Emerging models suggest that EVs participate in exercise-induced adaptations through the transfer of microRNAs, cytokines, and ROS-related signaling molecules [102,103].

### 7.2. Human Clinical Evidence

Clinical studies consistently demonstrate elevated levels of circulating EVs in individuals with obesity, prediabetes, T2DM, and associated cardiovascular disorders. Increased concentrations of specific EV subpopulations have also been reported in hypertension, chronic kidney disease, and heart failure, suggesting that EV alterations occur across multiple cardiometabolic conditions [104]. Notably, obese individuals without overt comorbidities exhibit elevated platelet-derived EVs that correlate with impaired fibrinolytic activity, indicating that EV abnormalities may arise early during disease development [97].

Circulating EVs have been strongly associated with endothelial dysfunction and vascular complications in patients with T2DM. Elevated endothelial-derived EVs correlate with impaired vasorelaxation, reduced flow-mediated dilation, and increased arterial stiffness, all of which are established predictors of cardiovascular risk [101]. Furthermore, EV-mediated inflammatory signaling contributes to chronic vascular injury and progression of diabetic complications.

Clinical observations also support a role for EVs as biomarkers of metabolic dysfunction. Changes in EV abundance and molecular cargo reflect ongoing alterations in insulin sensitivity, endothelial health, inflammation, and cardiovascular risk. Importantly, EVs derived from healthy endothelial cells contain biologically active eNOS that contribute to nitric oxide bioavailability, whereas this protective function is diminished in patients with endothelial dysfunction [25,69]. Such alterations in EV composition may therefore provide valuable insights into disease severity and progression.

Collectively, human studies suggest that circulating EVs serve not only as indicators of metabolic and vascular dysfunction but also as active contributors to disease pathogenesis. Their close association with obesity, insulin resistance, endothelial dysfunction, and cardiovascular complications supports their potential utility such as diagnostic biomarkers, prognostic indicators, and future therapeutic targets in T2DM.

## 8. EVs in Diabetic Complications

EVs have emerged as critical mediators in the pathogenesis of diabetes-related complications, including diabetic nephropathy (DN), diabetic cardiomyopathy (DCM), and DR. Abnormal EVs can transfer bioactive molecules such as miRNAs, mRNAs, and proteins between cells, thereby influencing intercellular signaling and exacerbating tissue injury in these complications [105].

### 8.1. Diabetic Nephropathy

Diabetic nephropathy, the leading cause of chronic kidney disease, is a microvascular complication marked by glomerular fibrosis, largely mediated by transforming growth factor-β1 (TGF-β1) [106]. Urinary EVs from DN patients exhibit elevated miR-320c, which downregulates thrombospondin 1 (TSP1) and accelerates fibrotic processes. Similarly, miR-192 in urinary EVs correlates positively with TGF-β1 expression, linking it to early DN development [105,107]. Glomerular endothelial cell-derived EVs from high-glucose conditions carry TGF-β1 mRNA that promotes proliferation, α-smooth muscle actin expression, and extracellular matrix production in glomerular mesangial cells via the TGF-β1/Smad3 pathway [108]. These EVs also induce epithelial–mesenchymal transition and glomerular barrier dysfunction in podocytes through the Wnt/β-catenin pathway, further contributing to renal fibrosis [109]. Collectively, these findings implicate abnormal EVs as key mediators in the progression of DN.

### 8.2. Diabetic Cardiomyopathy

Diabetic cardiomyopathy (DCM) is characterized by myocardial dysfunction secondary to metabolic dysregulation. EVs derived from diabetic cardiomyocytes contain elevated miR-320 and reduced miR-126 compared with controls. miR-320 can be transferred to cardiac endothelial cells, inhibiting proliferation, migration, and tube formation through downregulation of IGF-1, Hsp20, and Ets-2 [105,110]. Platelet EVs containing HSP70 fail to activate the ERK1/2–HSP27 pathway, reducing cardiomyocyte proliferation and angiogenesis [111]. Moreover, Mst1 protein in EVs from cardiac endothelial cells can be delivered to cardiomyocytes, where it suppresses autophagy, promotes apoptosis, and impairs glucose metabolism, further exacerbating DCM [112,113].

### 8.3. Diabetic Retinopathy

Diabetic retinopathy (DR) is a microvascular complication of advanced T2DM, marked by pericyte loss and aberrant angiogenesis. EVs from DR patients’ plasma can induce retinal microvascular dysfunction in experimental models. These EVs show elevated levels of miR-150-5p, miR-21-3p, and miR-30b-5p, which may contribute to pericyte detachment and pathological angiogenesis [114,115]. Additionally, miR-15a-enriched plasma EVs are taken up by Müller cells, inducing oxidative stress via Akt3 targeting and promoting retinal injury [105]. Pericyte-derived EVs also carry circRNA cPWWP2A, which can be transferred to endothelial cells to upregulate angiopoietin 1, occludin, and sirtuin 1 via miR-579 inhibition, leading to retinal vascular dysfunction [116].

## 9. A Unified Model of EV-Mediated Communication in Diabetes

Emerging evidence supports a unified model in which EVs act as key mediators linking metabolic dysfunction to the development of diabetic complications. In the initial phase, metabolic stress induced by hyperglycemia, lipotoxicity, oxidative stress, and chronic inflammation stimulates EV production from multiple cell types. As EV-mediated signaling spreads throughout the body, recipient endothelial and parenchymal cells undergo metabolic and inflammatory reprogramming, promoting endothelial dysfunction, oxidative stress, and vascular injury. Because the vasculature serves as a common target for circulating EVs, persistent EV-mediated communication may represent a central mechanism linking local metabolic disturbances to systemic disease progression.

During the second phase, stressed adipocytes, pancreatic β-cells, and immune cells release EVs enriched with bioactive cargo, including proteins, lipids, cytokines, and regulatory microRNAs. These EVs subsequently enter the circulation and contribute to the systemic propagation of pathological signals by modifying the function of distant target tissues.

**Figure 3:**
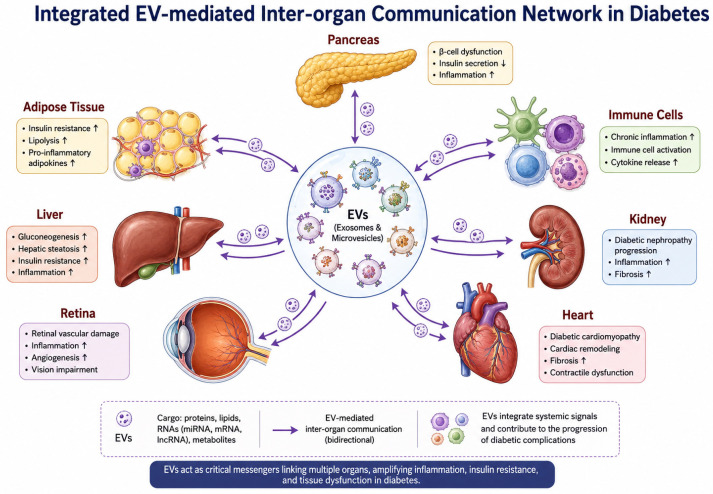
EV-mediated internal organ communication network in Diabetes.

Over time, cumulative vascular damage contributes to the development of major diabetic complications, including diabetic nephropathy, diabetic retinopathy, and diabetic cardiomyopathy. This framework provides a conceptual model that integrates findings from both T1DM and T2DM and highlights EVs as potential mechanistic links between metabolic stress, inter-organ communication, and chronic diabetic complications (Figure 3).

Importantly, this model is intended as a unifying conceptual framework based on accumulating experimental and clinical evidence. While numerous studies support individual components of this pathway, direct demonstration of a complete EV-driven inter-organ communication cascade in humans remains an active area of investigation.

## 10. EVs as Therapeutic Tools

Beyond their pathogenic roles, EVs have emerged as promising therapeutic agents for T2DM and associated complications. By delivering bioactive molecules such as proteins, miRNAs, and long non-coding RNA (lncRNAs), EVs can modulate target cell functions, ameliorating disease phenotypes. Importantly, EVs possess low immunogenicity and high tissue-targeting potential, making them attractive candidates for in vivo therapies [105].

### 10.1. Type 1 Diabetes Mellitus

While current treatments such as insulin therapy and glucose monitoring improve glycemic control, they do not fully prevent long-term complications. Pancreas and islet transplantation provide more definitive approaches but remain limited by immune rejection and poor graft survival. EVs offer a multifaceted therapeutic platform as they can enhance islet transplantation outcomes through angiogenesis and immune modulation, promote β-cell regeneration and differentiation, and serve as carriers for targeted delivery of protective RNAs or proteins. Continued research into the mechanisms of EV biology and their translation into clinical practice may ultimately transform both diagnostics and therapeutics in diabetes care [69,86,117].

### 10.2. Type 2 Diabetes Mellitus

Traditional T2DM management relies on hypoglycemic agents and insulin; however, EVs offer novel approaches for improving metabolic function. Adipose stem cell-derived EVs carrying STAT3 protein promote macrophage polarization toward an anti-inflammatory M2 phenotype via arginase transactivation, improving insulin sensitivity and metabolic balance in obese mice [118]. Bone marrow cell-derived EVs containing miR-106b-5p and miR-222-3p enhance pancreatic β-cell proliferation through downregulation of the Cip/Kip pathway [80]. Similarly, human MSC–derived EVs can alleviate peripheral insulin resistance and protect β-cells in diabetic rats, indicating a potential cell-free therapeutic strategy for T2DM [119].

### 10.3. Diabetic Nephropathy

Current DN treatments, including hemodialysis and kidney transplantation, are limited by cost and organ availability. MSC-derived EVs have demonstrated nephron protective effects by delivering renal trophic factors, exerting anti-apoptotic actions, and preserving tubular epithelial tight junctions [120]. Umbilical cord-derived EVs can overcome hyperglycemia-induced MSC dysfunction, enhancing their therapeutic efficacy [121]. MSC-EVs also promote autophagy via mTOR inhibition, mitigating renal fibrosis. EVs from human bone marrow MSCs and liver stem-like cells carry miRNAs that downregulate pro-fibrotic genes, significantly improving renal function in diabetic animal models [122].

### 10.4. Diabetic Cardiomyopathy and Retinopathy

While studies are limited, EVs show therapeutic promise in DCM and DR. HSP20-engineered EVs from transgenic cardiomyocytes can attenuate cardiac dysfunction, apoptosis, and fibrosis in diabetic mice, restoring cardiac function. EV-based therapies developed for other cardiovascular disorders, such as myocardial infarction and ischemic injury, suggest broader applicability [105]. In DR, EV-mediated delivery of protective molecules could similarly modulate retinal vascular function and prevent pericyte loss, although experimental evidence remains limited [123].

### 10.5. Diabetic Wounds

Delayed wound healing is a major complication in diabetes. Human fibroblast-derived EVs containing HSP90α, pro-angiogenic miRNAs (miR-126, miR-130a, miR-132), and anti-inflammatory miRNAs (miR-124a, miR-125b) promote angiogenesis, fibroblast activation, and keratinocyte migration. EVs from urinary stem cells enriched in DMBT1 enhance angiogenesis and accelerate wound repair [124]. Adipose-derived stem cell EVs overexpressing Nrf2 improve vascularization, reduce ROS and pro-inflammatory cytokines (IL-1β, IL-6, TNF-α), and support endothelial progenitor cell survival in high-glucose conditions [125]. EPC-derived EVs stimulate endothelial cell proliferation, migration, and angiogenesis via Erk1/2 signaling and upregulation of VEGF-A, VEGFR-2, and FGF-1 [126]. Platelet-rich plasma-derived EVs similarly promote wound closure via Rho-YAP pathway activation. These studies highlight EVs as potent modulators of diabetic wound repair [127].

## 11. Future Perspectives for EVs in Diabetes Mellitus

Despite significant advances in the field of EVs, several avenues remain to be explored to harness their potential in clinical practice fully. These aspects include.

### 11.1. EV Heterogeneity

The heterogeneity of EVs is related to their size, content, functional impact on recipient cells, and cellular origin. Size variability could be due to uneven invagination of the membranes of the MVB, leading to variable total content of fluid and solids. Detailed fractionation methods involving EVs showed that EVs may contain heterogeneous populations defined by a distinct size range. Size heterogeneity can also result in different amounts of EVs’ content. The milieu and the inherent biology of the cells may be reflected on the EVs’ content and their biological markers. EVs may have different types of cargos including various membrane, cytosolic and nuclear proteins, extracellular matrix proteins, metabolites, and nucleic acids (mRNA, noncoding RNA species, and DNA) [128].

### 11.2. Standardization and Characterization

A major challenge in EV research is the lack of standardized isolation and characterization protocols. Future efforts should focus on developing reproducible, scalable, and high-purity EV isolation techniques. Standardized characterization of EV size, cargo composition, surface markers, and bioactivity is essential to ensure comparability between studies and facilitate translation into clinical applications [129].

### 11.3. Mechanistic Insights

Although EVs have been shown to modulate inflammation, insulin resistance, endothelial dysfunction, and tissue repair, the precise mechanisms remain incompletely understood [130]. Future work should focus on defining how specific EV cargo (e.g., miRNAs, proteins, lipids) mediates intercellular communication and systemic effects. Investigating cell-type-specific EV signaling pathways in metabolic tissues, vascular endothelium, kidney, retina, and heart is required. Highlighting and elucidating EV interactions with immune cells and their contribution to chronic inflammation in DM.

### 11.4. Biomarker Development

EVs derived from plasma, urine, or other body fluids have shown promise as biomarkers for early detection, prognosis, and monitoring of DM and its complications. Future studies should aim to validate candidate biomarkers in large, multi-center cohorts to enhance clinical applicability. In addition to exploring the longitudinal changes in EV cargo to monitor disease progression or therapeutic responses. Creation and identification of a robust, reproducible panel of miRNAs, mRNAs, and proteins within EVs that predict disease onset and progression would be of use for a better understanding of their role and the potential therapeutic targets to be created.

### 11.5. Future Therapeutic Applications

Future research directions of EVs include engineering EVs with enhanced targeting, stability, and specific cargo to improve therapeutic efficacy and that requires better understanding of their contribution to the physiological and pathological processes. Moreover, conducting rigorous preclinical and early-phase clinical trials to assess safety, biodistribution, dosage, and long-term efficacy in humans. Also, considering the combination strategies with existing pharmacological treatments to achieve synergistic effects.

### 11.6. Personalized Medicine

EVs hold promise as a novel platform for personalized medicine in DM. Their molecular composition reflects the metabolic state and pathological context of the donor cells, enabling precision-tailored strategies. Therapeutically, EVs can be engineered or selected to match individual metabolic profiles and disease phenotypes, thereby enhancing treatment specificity. In parallel, longitudinal monitoring of circulating EV signatures may serve as a dynamic tool to guide therapeutic decisions and predict the onset or progression of complications. Moreover, integrating EV-based diagnostics with precision drug delivery systems could maximize therapeutic efficacy while minimizing adverse effects, offering a powerful approach to individualized diabetes management.

### 11.7. Integration with Emerging Technologies

Integration of EV research with emerging technologies has the potential to markedly accelerate their clinical translation in diabetes and related complications. High-throughput multi-omics profiling of EV cargo offers opportunities to uncover novel disease mechanisms and identify previously unrecognized therapeutic targets. In parallel, artificial intelligence and machine learning approaches can be leveraged to analyze complex EV signatures, enabling the prediction of disease risk, the stratification of patients, and the anticipation of therapeutic responses. Furthermore, advances in bioengineering have facilitated the development of EV mimetics and EV-inspired nanovesicles, which may address current challenges related to yield, stability, and tissue-specific targeting, thereby broadening the translational potential of EV-based strategies.

## 12. Current Limitations and Barriers to Clinical Translation of EVs in Diabetes

Despite the considerable promise of EVs as biomarkers and therapeutic agents in diabetes mellitus, several scientific, technical, manufacturing, and regulatory challenges continue to hinder their translation into routine clinical practice. Addressing these limitations is essential to ensure the reproducibility, safety, efficacy, and scalability of EV-based diagnostic and therapeutic strategies.

### 12.1. EV Heterogeneity

One of the most significant challenges in EV research is the inherent heterogeneity of EV populations. EVs differ substantially in their size, biogenesis, cellular origin, molecular composition, and biological activity. Even EVs isolated from the same cell type may exhibit distinct protein, lipid, and nucleic acid profiles depending on the physiological state of the donor cells, culture conditions, and environmental stimuli. [131]. In diabetes, factors such as hyperglycemia, oxidative stress, inflammation, obesity, and disease stage can profoundly alter EV cargo composition and biological function. Consequently, identifying reproducible EV signatures and establishing standardized biomarkers remain challenging [110]. This heterogeneity also complicates the development of EV-based therapeutics, as variations in cargo content may lead to inconsistent biological effects between studies and patient populations.

### 12.2. Isolation and Characterization Challenges

The lack of standardized isolation and characterization protocols remains a major obstacle in the EV field. Different isolation methods frequently yield vesicle populations with varying purity, composition, and functionality. Furthermore, characterization methods vary considerably among laboratories, resulting in inconsistencies in EV size measurements, particle concentrations, surface marker identification, and cargo profiling. This contributes substantially to inter-study variability and limits the reproducibility of reported findings [132,133].

### 12.3. Biodistribution and In Vivo Delivery

Efficient delivery of therapeutic EVs to target tissues remains another major translational barrier. Following systemic administration, a substantial proportion of EVs accumulate within the mononuclear phagocyte system, particularly in the liver and spleen. Hepatic uptake by Kupffer cells and splenic sequestration significantly reduce the amount of EVs reaching intended target organs, thereby limiting therapeutic efficacy [134].

Additionally, EV biodistribution is influenced by factors such as vesicle size, surface protein composition, route of administration, and recipient physiology [135]. In diabetes, disease-associated vascular dysfunction and altered tissue permeability may further affect EV trafficking patterns. Consequently, strategies to improve EV targeting, prolong circulation time, and minimize off-target accumulation remain active areas of investigation.

### 12.4. Targeting Specificity

Another major challenge is achieving precise delivery of EVs to specific tissues and cell populations. Native EVs possess some degree of tissue tropism determined by their surface proteins and lipid composition; however, this targeting is often insufficient for therapeutic purposes. Off-target uptake by non-diseased tissues may reduce treatment efficacy and increase the risk of unintended biological effects [136].

Emerging bioengineering approaches, including surface modification, ligand conjugation, genetic engineering of donor cells, and synthetic EV mimetics, are being explored to enhance tissue-specific targeting. Nevertheless, achieving highly selective delivery to pancreatic islets, adipose tissue, kidney, retina, or cardiovascular tissues remains a significant challenge in diabetes-focused EV therapeutics.

### 12.5. Storage Stability and Product Preservation

Long-term preservation of EV biological activity remains challenging. Storage conditions, freeze–thaw cycles, cryoprotectants, and storage duration can significantly affect EV integrity, membrane stability, cargo retention, and functional activity [137].

### 12.6. Safety Considerations

Although EVs are generally considered less immunogenic than cell-based therapies, important safety concerns remain [138]. Depending on their cellular origin, EVs may carry pro-inflammatory cytokines, pathogenic proteins, oncogenic nucleic acids, or other undesirable cargo capable of eliciting adverse biological effects.

In addition, repeated administration may induce immune responses, alter endogenous intercellular communication networks, or produce unanticipated off-target effects. Long-term safety data are currently limited, and comprehensive toxicological studies are required before therapeutic EVs can be widely adopted in clinical practice [139].

### 12.7. Cargo Loading Efficiency

For EV-based drug delivery applications, efficient loading of therapeutic molecules remains a significant technical challenge. Both endogenous loading approaches, which rely on donor-cell engineering, and exogenous loading methods, such as electroporation, sonication, extrusion, and chemical transfection, have limitations [140].

Moreover, achieving reproducible cargo loading while preserving EV functionality remains difficult. Improving loading efficiency and ensuring stable cargo retention will be crucial for the development of effective EV-based therapeutics [141].

### 12.8. Manufacturing and Scalability

The transition from laboratory-scale EV production to clinical-grade manufacturing presents significant challenges. Current production methods often generate insufficient EV quantities for large-scale therapeutic applications. Moreover, donor cell characteristics, culture conditions, passage number, and isolation procedures can all influence EV yield and composition [142].

Batch-to-batch variability remains a critical concern, particularly for therapeutic EV products. Even minor differences in manufacturing conditions may alter cargo composition and biological activity, potentially affecting therapeutic efficacy and safety [139]. Developing robust Good Manufacturing Practice (GMP)-compliant production pipelines, standardized quality control procedures, and scalable manufacturing technologies will be essential for future clinical implementation.

### 12.9. Regulatory and Standardization Challenges

The regulatory classification of EV-based products remains unclear in many jurisdictions. Depending on their source, manufacturing process, and intended application, EVs may be regulated as biological products, cell-derived therapeutics, advanced medicinal products, or drug delivery systems. This regulatory ambiguity complicates product development and clinical translation.

Furthermore, the absence of universally accepted standards for EV isolation, characterization, potency testing, and quality control creates additional challenges for regulatory approval. International efforts, including the Minimal Information for Studies of EVs (MISEV) guidelines, have improved reporting standards; however, further harmonization is needed before widespread clinical implementation can be achieved.

## 13. Conclusions

EVs have emerged as key mediators of intercellular communication in diabetes mellitus, influencing immune regulation, β-cell function, insulin resistance, endothelial dysfunction, and the development of diabetic complications. Evidence from both experimental and clinical studies supports a dual role for EVs as active participants in disease pathogenesis and as promising biomarkers that reflect disease progression and therapeutic responses.

Importantly, EVs provide a conceptual framework that links metabolic stress in the pancreas, adipose tissue, immune system, and vasculature to the development of diabetic nephropathy, retinopathy, cardiomyopathy, and other complications via systemic inter-organ communication. This integrated perspective highlights EVs as potential mechanistic drivers of disease progression across multiple organs.

Beyond their pathogenic roles, EVs hold considerable promise as minimally invasive biomarkers and as therapeutic platforms for targeted drug delivery, immune modulation, tissue repair, and regenerative medicine. However, significant challenges—including EV heterogeneity, lack of standardized isolation methods, limited targeting specificity, manufacturing scalability, and regulatory hurdles—must be addressed before widespread clinical implementation can be achieved.

Overall, EVs represent a rapidly advancing field with the potential to transform our understanding of diabetes biology and to enable novel diagnostic and therapeutic strategies. Continued mechanistic studies, technological innovations, and well-designed clinical trials will be essential to realize the full translational potential of EV-based approaches in diabetes and its complications.

## Figures and Tables

**Table 1:** Summary of the methods of EV isolation and purification.

Method	Principle	Advantages	Disadvantages
Differential Ultracentrifugation	Sequential centrifugation at increasing speeds to pellet progressively smaller particles.	Widely used; cost-effective.	Time-consuming; risk of contamination from debris/apoptotic bodies.
Density-Gradient Separation	Uses density media (e.g., sucrose, iodixanol) to separate vesicles by buoyant density.	High purity; preserves EV integrity and bioactivity.	Labor-intensive; lower throughput.
Size Exclusion Chromatography (SEC)	Runs sample through porous stationary phase, separating by particle size.	Gentle method; reproducible; effective for blood plasma.	May co-isolate non-EVs’ particles; limited scalability.
Immunological Isolation (IAC)	Antibody-coated beads capture EVs based on surface markers.	Highly specific; can isolate subpopulations.	Expensive; requires known markers.
Precipitation	Uses polymers (e.g., PEG) to aggregate and precipitate EVs.	Simple; scalable.	Lower purity; risk of co-precipitating contaminants.
Tangential Flow Filtration (TFF)	Membrane-based continuous filtration.	Scalable, GMP-compatible, high recovery.	Requires specialized equipment.
Microfluidic Isolation	Separation based on size, affinity, or flow dynamics.	Rapid, low sample volume, high specificity.	Limited throughput, expensive.
Asymmetric Flow Field-Flow Fractionation (AF4)	Separation of particles in a laminar flow channel using a perpendicular crossflow field, based on hydrodynamic size and diffusion properties.	High-resolution separation; label-free; preserves EV integrity; enables fractionation of EV subpopulations and separation from protein aggregates/lipoproteins.	Requires specialized instrumentation and expertise; relatively low throughput; method optimization can be complex.

**Table 2:** Summarizes the most commonly used techniques for EV quantification.

Technique	Principle	Advantages	Limitations
Nanoparticle Tracking Analysis (NTA)	Tracks the Brownian motion of individual particles to calculate size via the Stokes–Einstein equation.	Measures individual particles; real-time visualization; minimal sample prep.	Reduced sensitivity for very small particles; accuracy decreases for larger particles due to slower motion and viscosity effects.
Vesicle Flow Cytometry	Detects light scatter and fluorescence of particles passing through a laser.	High-throughput; adaptable from standard cytometers; can use fluorescence labeling.	Limited detection of the smallest vesicles; size distribution skewed toward larger particles; optical property variations complicate size correlation.
Surface Plasmon Resonance (SPR)	Detects binding of EVs to functionalized surfaces (e.g., antibodies).	Sensitive to binding events; potential for specific detection.	Relies on binding, which can bias quantification; standardization is challenging; not yet widely available.
Transmission Electron Microscopy (TEM)	Transmits an electron beam through a thin specimen to visualize internal ultrastructure and morphology of EVs.	Gold standard for EV morphology; nanometer-scale resolution; enables visualization of vesicle size and membrane structure.	Extensive sample preparation may introduce artifacts (e.g., dehydration, cup-shaped morphology); labor-intensive; limited quantitative capability.
Scanning Electron Microscopy (SEM)	Scans the sample surface with a focused electron beam to generate high-resolution images of surface topology.	Provides detailed three-dimensional surface morphology; useful for assessing EV shape and aggregation.	Lower resolution for internal structures; requires conductive coating and extensive sample preparation; potential sample distortion.
Cryo-Electron Microscopy (Cryo-EM)	Images rapidly frozen specimens in a near-native hydrated state without fixation or staining.	Preserves native EV morphology and ultrastructure; minimizes preparation artifacts; provides high-resolution structural information.	Expensive instrumentation; technically demanding; low throughput; requires specialized expertise and data analysis.

## Data Availability

No new data were created or analyzed in this study.
